# Advances in the study of autophagy in breast cancer

**DOI:** 10.1007/s12282-023-01541-7

**Published:** 2024-02-05

**Authors:** Tang Yu, Liu Rui, Zhao Jiumei, Li Ziwei, Hu Ying

**Affiliations:** 1 The Second Affiliatied Hospital of Kunming Medical University and Department of Clinical Larboratory, Kunming, China; 2 The Third Affiliated Hospital of Kunming Medical University, Kunming, China; 3 The Third Affiliated Hospital of Chongqing Medical University, Chongqing, China; 4 Chongqing Nanchuan District People’s Hospital, Chongqing, China; 5 Chongqing Health Center for Women and Children, Women and Children’s Hospital of Chongqing Medical University, Chongqing, China

**Keywords:** Autophagy, Breast cancer, Molecular mechanism, Autophagy inhibitor, Therapy

## Abstract

Breast cancer is the most prevalent malignant tumor among women, with a high incidence and mortality rate all year round, which seriously affects women's health. Autophagy, a well-conserved cellular process inherent in eukaryotic organisms, plays a pivotal role in degrading damaged proteins and organelles, recycling their breakdown products to aid cells in navigating stress and gradually restoring homeostatic equilibrium. Recent studies have unveiled the intricate connection between autophagy and breast cancer. Autophagy is a double-edged sword in breast cancer, demonstrating a dual role: restraining its onset and progression on one hand, while promoting its metastasis and advancement on the other. It is also because of this interrelationship between the two that regulation of autophagy in the treatment of breast cancer is now an important strategy in clinical treatment. In this article, we systematically survey the recent research findings, elucidating the multifaceted role of autophagy in breast cancer and its underlying mechanisms, with the aim of contributing new references to the clinical management of breast cancer.

## Introduction

Breast cancer stands as the most prevalent malignancy tumor among women, and as per the latest global statistics from 2020, it has eclipsed lung cancer to become the most commonly diagnosed cancer worldwide. Among the 14 most common cancers, breast cancer has witnessed the most significant increase in mortality rates and currently ranks as the fourth leading cause of death globally, posing a grave threat to women’s health on a global scale [1]. Presently, the primary therapeutic modalities for breast cancer are radiotherapy, chemotherapy, molecular-targeted therapy and endocrine therapy [2]. Nevertheless, the emergence of resistance in cancer cells to these conventional treatments has emerged as a major challenge in the management of breast cancer. Autophagy, an evolutionarily conserved process widely distributed in eukaryotic cells, entails the intracellular engulfment and degradation of cellular structures via lysosomes. This process is instrumental in regulating cellular metabolism and influencing a wide array of physiological and pathological processes in organisms [3,4]. Aberrations or mutations in autophagy pathways have been linked to various human diseases, including cardiovascular disorders, metabolic diseases, neurological diseases and cancer [5,6]. Autophagy exhibits a dual role in the development of tumors. On one hand, in the early stages of cancer, autophagy exerts tumor-suppressive effects by curbing gene mutations and the generation of reactive oxygen species (ROS) [7]. On the other hand, autophagy promotes tumor development by facilitating tumor angiogenesis, removing and recycling damaged intracellular proteins and organelles, and providing a conducive environment for the survival of tumor cells [6]. Notably, autophagy can promote tumor cell resistance to conventional treatments such as radiotherapy, with some evidence suggesting [8] that chemotherapy induces cellular autophagy, potentially one of the main mechanisms contributing to drug resistance in cancer cells. Therefore, scientists proposed that chemotherapy effects might be enhanced by inhibiting autophagy, leading to many experimental studies assessing autophagy inhibitor and drug combinations.

In recent years, with the increasing research on autophagy, a large number of findings have shown that autophagy is closely related to breast cancer, and the alteration of autophagy function affects the process of breast cancer development, which has opened new avenues for basic breast cancer research. Autophagy emerged as a multifaceted and dynamic element in breast cancer, and current studies have revealed diverse mechanisms of its role in breast cancer, and many studies have demonstrated a direct involvement in the progression of the disease. However, despite this growing body of evidence, a comprehensive review that consolidates and synthesizes these findings is conspicuously absent. Therefore, this review seeks to provide a comprehensive analysis of recent reports regarding the interplay between autophagy and breast cancer. It aims to shed light on the intricate relationship, underlying mechanisms and its clinical implications. Ultimately, this endeavor is aimed to provide a solid theoretical foundation for both basic research and clinical treatment in the realm of breast cancer.

## Autophagy overview

Cellular autophagy is a ubiquitous self-regulatory within eukaryotic cells, serving as a vital response to challenging environments such as starvation, hypoxia and energy stress states [9–11]. This intricate process encompasses a form of physiological cell death, commonly known as type II programmed cell death [12]. Its diverse physiological functions encompass the removal of malfunctioning or damaged cells, the maintenance of internal environmental homeostasis, the induction of cell death, the regulation of immune function, and participation in extending cell lifespan. According to the lysosomal pathway, autophagy can be categorized into three primary types [13]: (i) Macroautophagy: This process involves the formation of autophagosomes, which are double-membraned structures that encapsulate cytoplasmic proteins and organelles. These autophagosomes ultimately fuse with lysosomes, forming autophagic lysosomes that degrade their contents. (ii) Microautophagy: This form entails the direct invagination of lysosomal or endovollicular membranes into the cytoplasm for degradation. (iii) Molecular chaperone-mediated autophagy: This mechanism is exclusive to mammalian cells, where intracytoplasmic proteins first bind to molecular chaperones and are subsequently translocated into the lysosomal lumen for degradation [14]. In most contexts, when referring to cellular autophagy without specific distinction, it generally refers to macroautophagy. The occurrence of cellular autophagy can be divided into four distinct stages: autophagic vesicle precursor formation, auto phagosome membrane formation and extension, lysosome closure and fusion, and intracapsular degradation [14]. While the majority of our understanding of autophagy has been gleaned from studies in yeast, where approximately 30 autophagy-related genes have been identified, it is important to note that the autophagic process involves a complex interplay of signaling molecules and pathways [15].

The initiation phase of cellular autophagy primarily revolves around key proteins, namely ULK1, FIP200, and autophagy-related genes (Atg)13 [16]. In a well-nourished cellular environment, the mammalian target of rapamycin complex 1 (mTORC1) becomes active, resulting in the extensive phosphorylation of Atg13 and ULK1. This, in turn, serves to inhibit the onset of cellular autophagy [17,18]. In stark contrast, under conditions of nutrient scarcity, mTORC1 undergoes dephosphorylation of Atg13, which subsequently associates with and activates ULK1. This activated ULK1 then forms dimers, culminating in the initiation of cellular autophagy, with FIP200 playing a vital role in this process [19]. Furthermore, it has been shown that the formation of autophagosomal bilayers requires the participation of molecules such as phosphatidylinositol kinase P150 and Beclin-1. At this critical stage, the microtubule-associated protein LC3 emerges as a key determinant of autophagic size and serves to facilitate the bending of membranes during autophagosome formation [20].

The extension phase of autophagy primarily involves a cadre of molecules, including Atg12, LC3 (a yeast homolog of Atg8), Atg10, Atg16, Atg5, Atg7, Atg3, Atg4, and more [21]. The process is similar to the E1-E2-E3 ubiquitination process and can be subdivided into two ubiquitination-like processes: the binding of Atg12 and the modification of Atg8 [22]. During this phase, the Atg4 protease shears the C-terminus of the LC3 protein precursor, converting Atg8 into soluble LC3-I. Subsequently, Atg7 acting as an E1-like enzyme, facilitates the transports of LC3-I to Atg3, functioning as an E2-like enzyme, for covalent binding to phosphatidyl. Atg7 also transports Atg12 to Atg10, another E2-like enzyme, which covalently links Atg12 to Atg5. This interaction leads to the subsequent binding of Atg16, forming the Atg12-Atg5-Atg16 complex. This complex serves as a critical regulator for the extension of the autophagosomal membrane [23].

During the maturation stage, autophagosomes undergo fusion with lysosomes to form mature autophagic lysosomes. Key molecules central to this stage include LAMP1, LAMP2, UVRAG and monomeric GTPases [24]. The degradation of autophagosomal membranes is initiated by p62, which directly interacts with ubiquitination-related proteins and LC3, facilitating their roles within the autophagic process [25]. This process may also have the involvement of microtubule backbone proteins within the autophagosome. However, the precise details of this process and the underlying mechanisms remain to be further elucidated.

In the degradation phase, the encapsulated material within autophagic lysosomes is broken down by hydrolases, and the resulting small molecule degradation products are subsequently translocated back into the cytoplasm for cellular reuse. The initiation of the degradation process is mainly achieved through the activation of cathepsins B, D and L [26]. This intricate process encompasses the involvement of Atg12 and Atg15, which mediate the rupture of autophagic lysosomal membranes. Additionally, Atg1 and Atg13 play pivotal roles in facilitating the transportation of hydrolases within lysosomes, while the transport of some of the amino acids generated by degradation may also entail the participation of Atg22 [27].

## The relationship between autophagy and tumors

In different stages of tumor development and under varying environmental conditions, autophagy plays different roles, which can be categorized into the oncogenic role of autophagy and the pro-cancer role of autophagy.

The suppressive role of oncogenesis of autophagy is demonstrated by its capacity to eliminate damaged organelles and proteins, control inflammatory responses, protect gene stability to maintain cellular homeostasis and restrain tumor cell dissemination and invasion from their primary sites [28]. In addition, when certain autophagy-related proteins are abnormally expressed, they hinder the initiation of autophagy, thereby promoting tumor growth, proliferation, dissemination and invasion. For instance, Beclin 1, a key protein involved in autophagosome formation, has exhibited reduced expression in patients afflicted with breast, cervical, and pancreatic cancers. This decline in Beclin 1 levels suggests that inhibition of autophagy may enhance tumor growth and proliferation [29–31]. Furthermore, research involving mice has demonstrated that those with copy number deletions of the becn1 regulatory gene exhibited a higher propensity for spontaneous tumorigenesis compared to their wild-type counterparts [32]. Therefore, it is postulated that autophagy plays an inhibitory role in tumorigenesis especially in the initiation phase of tumors. This inhibitory effect is likely mediated through the following mechanisms: (1) Autophagy can inhibit mutations in genes and chromosomes through various mechanisms, thus reducing the possibility of cells undergoing malignant transformation. (2) Reactive oxygen species are highly genotoxic, and autophagy can inhibit the production of reactive oxygen species and the aggregation of cheesy proteins formed during redox reactions, primarily by clearing dysfunctional mitochondria.

The pro-cancer effect of autophagy becomes evident as tumors progress and continue to divide and proliferate, particularly in the face of challenging environments like starvation or oxygen deprivation. This phenomenon is frequently observed in tumor cells residing in poorly vascularized regions within solid tumors, where autophagy serves to enhance the growth and survival of tumor cells [33]. In conditions of chronic hypoxia and nutrient deficiency, often experienced by tumor cells inadequate blood supply, autophagy emerges as a vital mechanism for recycling waste materials to sustain essential cellular functions for cell survival [34]. As tumors advance to later stages, characterized by metabolic stress or genotoxicity, they activate autophagic mechanisms, and in some cases, even promote cell metastasis to maintain survival [35]. For example, heightened expression of hypoxia-inducible factor 1α (HIF1α) can stimulate autophagy, angiogenesis and the induction of BNIP3. Hypoxia leads to mitochondrial damage and the accumulation of intracellular reactive oxygen species. In this hypoxic milieu, BNIP3 takes charge of regulating intracellular autophagy, leading to the clearance of damaged intracellular mitochondria through autophagic mechanisms, thereby supporting tumor cell survival [36]. Studies in hepatocellular carcinoma have shown that the deletion of Atg7 or Atg5 resulted in benign hepatocellular carcinoma as opposed to its malignant counterpart [37]. Moreover, in human breast cancer, tumorigenic and invasive tumor epithelial cells preexist in intraductal carcinoma in situ, and the survival of these cells requires intracellular autophagy [38].

**Fig. 1:**
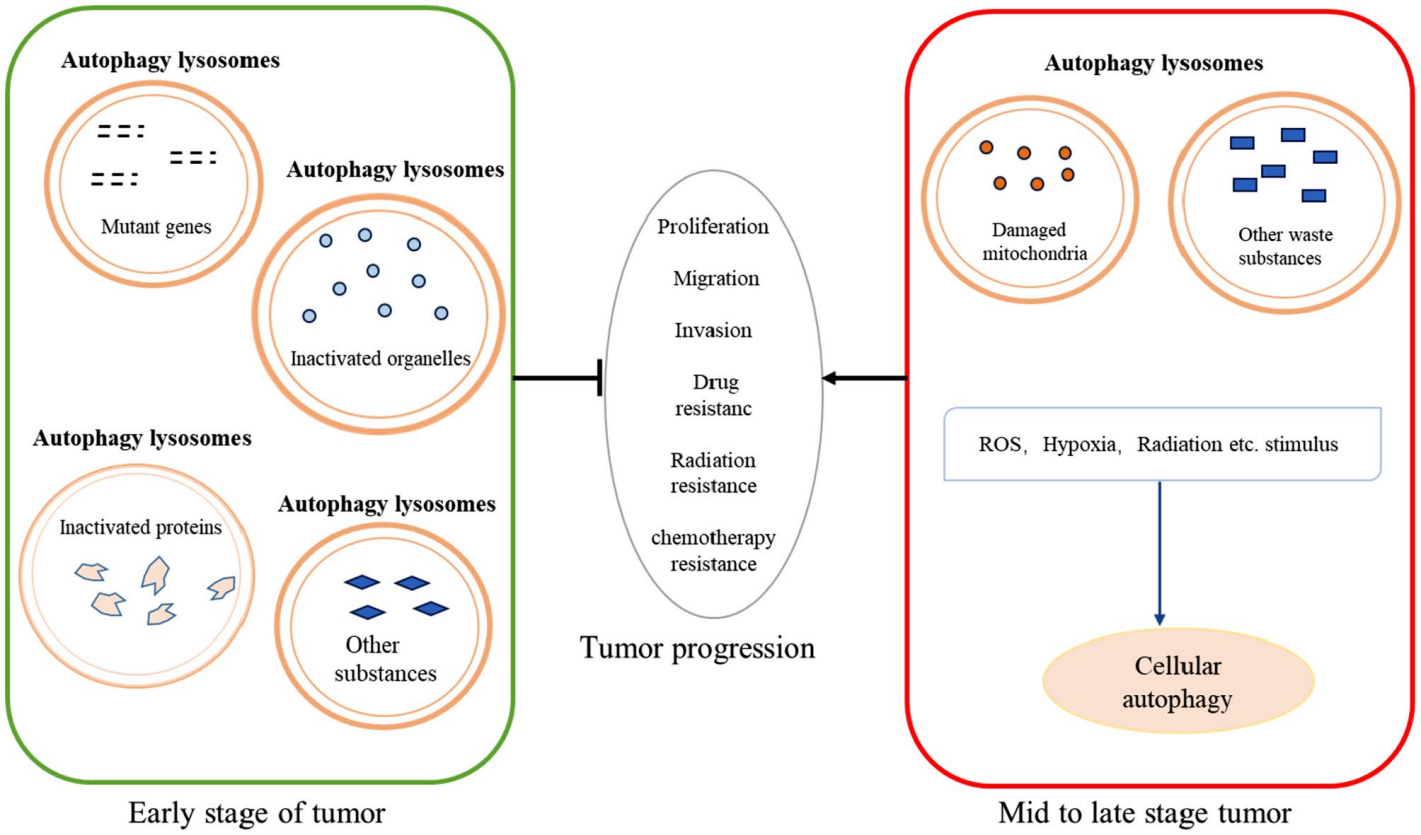
Effect of autophagy on physiological function of tumors

The above results indicate that autophagy is a double-edged sword. In the early stages of tumor development, autophagy serves as a protective factor to prevent tumor development and stabilize the normal physiological functions of cells by removing mutated genes and inactivating organelles. However, as tumors progress into the middle and late stages, characterized by heightened cell proliferation, enhanced migration, and other functional adaptations, tumor cells demand substantial amounts of energy and resources to facilitate their survival. In this context, autophagy plays a distinct role, enabling tumor cells to create a favorable environment for their survival (Fig. 1).

## Autophagy and breast cancer

As one of the common malignancies in women, the development of breast cancer is also associated with autophagy, and Beclin-1 gene is a hot gene in both autophagy research and the breast cancer field. Multiple studies have revealed that Beclin-1 gene plays a multifaceted role in breast cancer. It not only reduces the risk of breast cancer by acting as a tumor suppressor but also regulates autophagy to promote breast cancer development [29,39,40]. An analysis of breast cancer tissues has shown that approximately 70% of specimens exhibit reduced protein expression levels of Beclin-1 [41]. Moreover, given its proximity to the chromosome, Beclin-1 is often subjected to common deletion along with the breast cancer susceptibility gene 1 (BRCA1), making Beclin-1 deletion more likely to induce breast cancer [42]. The expression level of Beclin-1 in breast cancer cells was detected by Choi et al., which found that the negative rate of Beclin-1 was 70.1%, the weak positive rate was 26.2%, and the positive rate was only 3.7% [43]. Moreover, the expression level of Beclin-1 is also affected by different subtypes of breast cancer, with higher levels observed in more aggressive types such as human epidermal growth factor receptor type 2 (HER2) positive and basal-like (mostly triple-negative) breast cancers compared to the less malignant intraluminal A/B (luminal A/B) subtype [44]. The overexpression of the Beclin-1 gene in breast cancer MCF-7 cells has been found to enhance autophagic activity, subsequently inhibiting cell proliferation and tumor formation in nude mice [45]. It was found that in Beclin-1 gene-deficient breast cancer MDA-MB-231 cells could be detected, the phosphorylation levels of ERK and AKT were increased in breast cancer cells, Beclin-1 was negatively correlated with the intensity and duration of growth factor receptor signaling pathway persistence. Beclin-1 also demonstrated the ability to restrain the invasive metastasis of breast cancer cells [46]. These studies collectively suggest that reduced expression levels of Beclin-1 gene may inhibit the autophagic activity of breast cancer cells to promote the progression of breast cancer. However, as research advanced, a more complex picture emerged, demonstrating that Beclin-1 could promote breast cancer progression, even when highly expressed in some patients. Cha et al. reported that in a study of 692 breast cancer patients with invasive ductal carcinoma (IDC) and 114 invasive lobular carcinoma (ILC), the expression level of Beclin-1 was higher in IDC cases with higher proliferative activity [47]. In a pilot study, the culture of breast cancer MDA-MB-231 and BT-549 cells under normal conditions and transfection of the Beclin-1 gene resulted in inhibited cell growth and proliferation. However, when these cells were cultured under adverse conditions like hypoxia and nutrient deficiency, transfection of the Beclin-1 gene led to reduced cell death [48,49]. This could be attributed to the heightened autophagic activity in MDA-MB-231 and BT-549 cells following Beclin-1 gene transfection, providing a mechanism by which breast cancer cells reduce their metabolic stress and thus promote their survival in harsh environments such as starvation and hypoxia. The above studies suggest that Beclin-1 may have different effects on breast cancer cells in different environments, Nevertheless, the intricate mechanisms through which Beclin-1 affects both sides of breast cancer progression require further investigation.

In addition to Beclin-1, several autophagy-related genes are also involved in autophagy formation, such as ATG8 and resistance to ultraviolet-associated radiation gene (UV RAG). These genes are often detected as locus deletions or mutations in breast cancer patients [50]. Such findings may suggest that these genes serve as inhibitors of tumor progression by regulating autophagy. Organismal tissues under hypoxia can induce autophagy in tumor cells through the mediation of HIF1α [51]. It has been found that in tumor cells HIF1α can help tumor cells survive in a hypoxic environment by influencing cellular metabolism, promoting invasive dissemination, modulating pH, and impacting inflammation. In the context of breast cancer cells, the activation of Peroxisome Proliferator-Activated Receptor γ (PPARγ) triggers an upregulation of HIF1α expression, and HIF1α, in turn, mediates PPARγ-induced activation of autophagy, thereby promoting the survival of MDA-MB-231 breast cancer cells [52]. Under conditions of starvation and hypoxic, autophagy can be induced by activating Adenylate-Activated Protein Kinase (AMPK) or inhibiting the mTOR pathway. Additionally, in MCF7 and MDA-MB-231 breast cancer cells, autophagy is also involved in endoplasmic reticulum protein degradation to promote apoptosis in breast cancer cells [53].

Lamin A/C is one of the main components of nuclear fibrillar layer proteins that provide stability to the nucleus and establish a conducive environment for stable gene expression. Recent discoveries indicate that in the presence of DNA damage, Lamin A/C undergoes ubiquitination-like modifications and is subsequently degraded by the autophagic lysosomal pathway through binding to LC3. This process results in nuclear leakage and the demise of triple-negative breast cancer cells [54]. Moreover, studies have revealed that in the absence of autophagy, excessive accumulation of TNC proteins hinders T cell activation, leading to reduced T cell infiltration within triple-negative breast cancer tissues. This creates conditions conducive to unchecked tumor cell growth and development, thereby promoting the progression of breast cancer [55]. These two studies suggest that autophagy appears to be an important oncogenic factor in breast cancer, with its absence facilitating tumor cell survival.

**Fig. 2:**
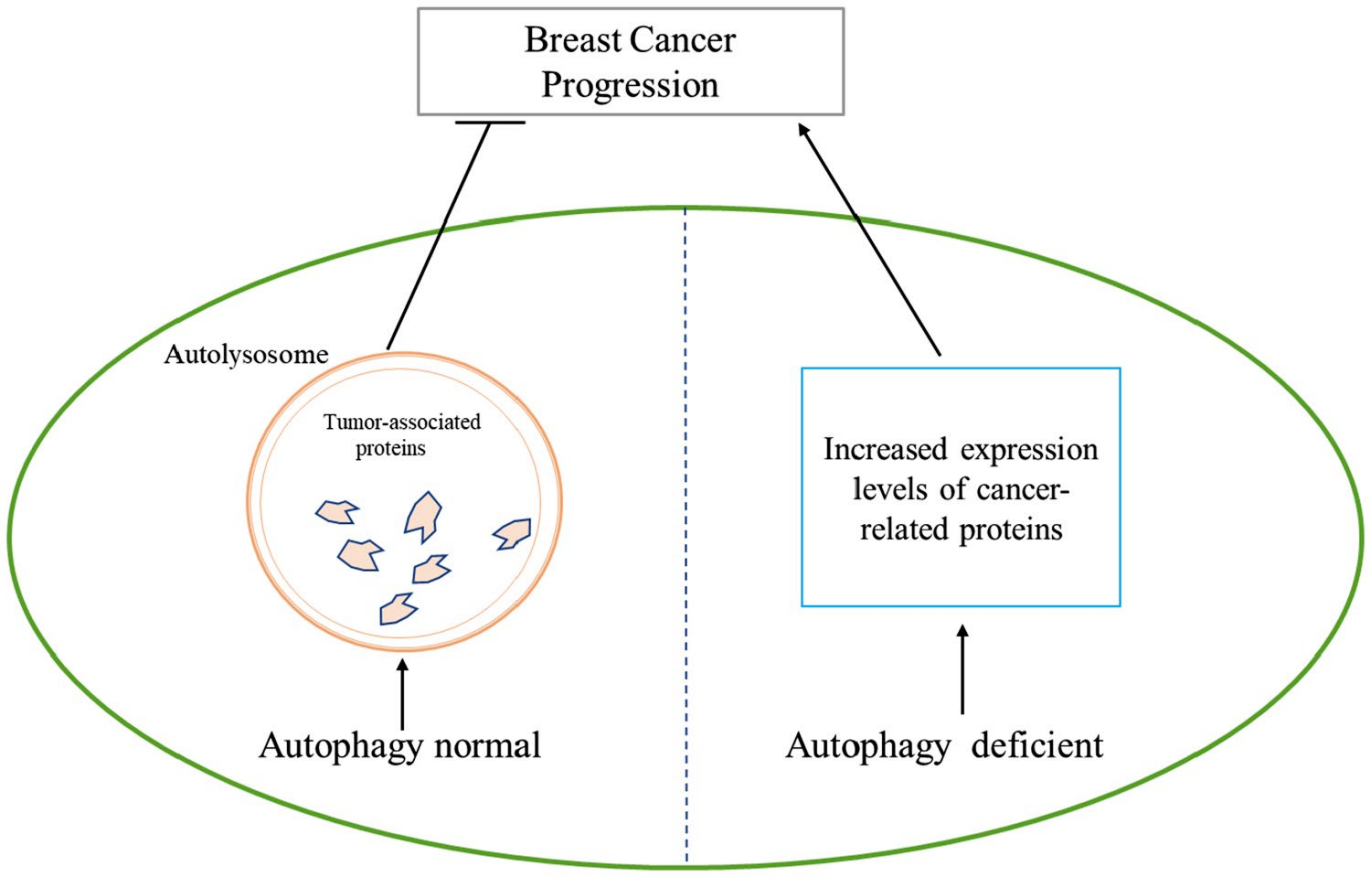
Effect of autophagy deficiency or normal status on breast cancer progression

The above findings suggest that autophagy also seems to have a dual role in breast cancer, with a predominant expression of its oncogenic role, Autophagy tends to function as a protective mechanism for breast cancer cell survival primarily when these cells are exposed to external stimuli (see Fig. 2). Autophagy is an unstable factor with very broad effects in cells, therefore, revealing the specific mechanism of autophagy's role in breast cancer to maintain it in a direction favorable to patient treatment could be very helpful for the prognosis of clinical breast cancer patients.

## Study of autophagy and cancer-related pathways in breast cancer

Breast cancer development is a complex process that is accompanied by the activation of many cancer-related pathways. Several studies have reported that the activation of various signaling pathways in breast cancer is closely interconnected, either directly or indirectly, with autophagy.

One of the most commonly studied pathways associated with autophagy is the AKT-mTOR signaling pathway. For instance, Abdullah et al. found that eugenol could induce autophagy and apoptosis in breast cancer cells by inhibiting the PI3K/AKT/FOXO3a pathway [55]. In a recent study, it was observed that Chaga mushroom extract (CME) inhibited the proliferation of 4T1 mouse breast cancer cells in a dose- and time-dependent manner. CME treatment led to increased phosphorylation of LC3 and AMPK while inhibiting the phosphorylation of S6 and S6K1 of mTOR. These results suggest that CME induces autophagy by activating the AMPK pathway and inhibiting the mTOR signaling pathways, ultimately inhibiting tumor cell proliferation [56]. Furthermore, it was also found that the combination of doxorubicin (DOX) and magnoflorine (Mag) inhibited the proliferation and migration of breast cancer, acting by suppressing autophagy induced by mTOR-dependent signaling pathway [57]. These findings suggest that in breast cancer, alterations in tumor cell function resulting from external environmental factors can be displayed through the AKT-mTOR-autophagy axis.

In the context of breast cancer, besides the AKT-mTOR signaling pathway, the NF-κB signaling pathway also seems to have some associativity with autophagy. Notably, it was found that miRNA-1910-3p within exosomes promotes breast cancer proliferation, metastasis, and autophagy by targeting MTMR3 and activating the NF-κB signaling pathway [58]. Additionally, polyphenol D has been found to induce apoptosis and protective autophagy in breast cancer cells through the JNK1-Bcl2 signaling axis [59]. These findings suggest that in breast cancer cells, immune and apoptosis-related signaling pathways are also involved in cellular autophagy, finally affecting the function of tumor cells.

The studies outlined above demonstrate that in breast cancer, autophagy is not only influenced by tumor-related signaling pathways but also has the capacity to affect the activation status of certain tumor signaling pathways. For example, it was found that the expression of WNT pathway-related proteins was suppressed after the knockdown of ATG4A, a key regulator of cellular autophagy processes [60]. This suggests that the activation of the WNT pathway seems to have a linkage with autophagy in breast cancer cells as well.

**Fig. 3:**
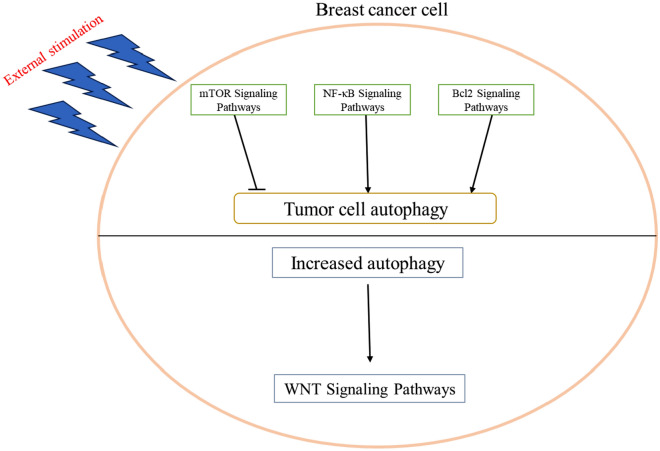
Association between autophagy and some tumor-associated signaling pathways in breast cancer

Taken together, these findings suggest that in breast cancer cells, various pathways can regulate the autophagic function of cells, most of which are stimulated by the external environment and eventually affect the physiological function of cells through autophagy. Meanwhile, certain pathways are regulated by autophagy and finally affect cellular physiological functions through various signaling pathways (Fig. 3).

## Autophagy and clinical translational research

In recent years, the close relationship between autophagy and breast cancer has been continuously discovered, and autophagy plays an important role in the treatment of breast cancer. The therapeutic strategies for most cancers, including breast cancer, predominantly involve the activation of apoptotic signaling to induce tumor cell death. The signals of apoptosis and autophagy are coupled to each other within cells, and these two pathways are closely intertwined and mutually influential. Therefore, the activation or inactivation of apoptosis can significantly impact the status of autophagy [61]. According to the dual nature of autophagy on tumor development, the treatment of breast cancer can be approached by either promoting autophagy to induce cell death or inhibiting autophagy when it exhibits cytoprotective effects. Autophagy can be effectively induced through the application of anticancer drugs and some autophagy inducers, including radiotherapy, ultimately leading to cell death.

Some studies have shown that specific drugs with autophagy-inducing effects have a potential anticancer therapeutic efficacy by enhancing cell death [62]. For example, Cryptotanshinone has been shown to promote the formation of vesicles in MDA-MB-231 breast cancer cells, inducing autophagy, and subsequently inhibiting migration, invasion, and proliferation of cancer cells [63]. Another study revealed that miR-199a-5p overexpression in breast cancer cells could upregulate the expression of autophagy-related genes BECN1 and DRAM1, consequently activating cellular autophagy. This activation notably enhanced the ability of ionizing radiation to induce cell death in cancer cells [64]. Collectively, these studies suggest that harnessing autophagy to induce cell death represents a promising and innovative approach to the treatment of breast cancer.

Resistance of breast cancer cells to therapy remains a major challenge in disease management, and autophagy emerges as a pivotal factor influencing tumor drug resistance. Particularly in the context of targeted therapy for HER2-positive breast cancer, drug resistance caused by autophagy is more serious when using the monoclonal antibody Herceptin and epidermal growth factor receptor tyrosine kinase inhibitors like lapatinib [65]. Research indicates that the level of autophagy in tumor cells influences their resistance to therapy. In breast cancer patients, once the tumor develops, tumor cells respond to treatment by upregulating autophagy, promoting cancer cell survival in treatment-induced stress, ultimately triggering drug resistance [66]. In a recent clinical study, it was found that simultaneous administration of autophagy inhibitors, like chloroquine, alongside targeted therapy in breast cancer patients can enhance tumor cell death, signifying a potential breakthrough in treatment [67]. Numerous studies have demonstrated that the combination of chloroquine with conventional therapies enhances the overall antitumor effect [68–72]. However, it was previously suggested that the occasion of chloroquine treatment of breast cancer may not be related to the inhibition of autophagy. Experimental studies have shown that when using DNA damage drugs such as cisplatin or drugs targeting autophagy regulators like Pt-dIns3K inhibitor LY294002 and mTOR inhibitor rapamycin acting on breast cancer cells 67NR and 4T1 in mice, can enhance the sensitivity of cancer cells to these drugs when chloroquine is applied in combination [73]. However, chloroquine did not enhance the efficacy of these drugs without knockdown of Atg12 and becn-1 or inhibition of autophagy using bafilomycin or even in the absence of Atg12 [74]. Therefore, the investigators speculated that the mechanism through which chloroquine enhances its effectiveness in combination with other breast cancer therapeutic agents may not be due to its inhibition of autophagy. This different conclusion needs to be further explored. Additionally, Zhou et al. found that the application of MK-8776, a cell cycle detection site kinase 1 inhibitor, in human triple-negative breast cancer cells increased the sensitivity of cancer cells to radiotherapy by inhibiting autophagy [75]. These studies highlight that the treatment of breast cancer can also be achieved by inhibiting autophagy, which has cytoprotective effects.

Cancer stem cells (CSCs) have the general properties of stem cells, including self-renewal and differentiation abilities, which can influence the process of tumor recurrence, metastasis and therapeutic response, contributing significantly to the heterogeneity observed in most tumors [76]. In the context of breast cancer, breast cancer stem cells (BCSCs) play a pivotal role in tumor development, therapy outcomes, and prognosis. BCSC-enriched populations of breast cancer cells show significant resistance to conventional chemotherapy [77]. Recent studies have shown that autophagy plays a crucial role in tumor stem cell genesis, maintenance and tissue metastasis. The overexpression of acetaldehyde dehydrogenase (ALDH) has an important impact on tumor invasion, metastasis and overall prognosis. ALDH1, highly expressed in all types of breast cancers, predicting a poor prognosis and heightened metastatic potential [78]. Distinct breast cancer subtypes display varying expression levels of three essential BCSC markers: CD44, CD24, and ALDH [79]. Notably, a high phenotypic ratio of CD44 + /CD24– /low in triple-negative breast cancer cells suggests a poor prognosis [80]. Autophagy contributes to the maintenance of BCSC properties by regulating CD24 and interleukin-6 (IL-6) secretion. The IL-6/STAT3 pathway has been found to play an important role in the survival process of BCSCs and in xenografts of triple-negative breast cancer [81]. Inhibition of the autophagy regulator FIP200 in BCSCs reduces its capacity to induce tumor formation by regulating the STAT3 and TGFβ/Smad pathways [82]. Hypoxia-induced autophagy can cause chemotherapy resistance in BCSCs, resulting in the development of drug resistance. However, autophagy inhibitory drugs like chloroquine (CQ) or the knockdown of autophagy-related genes can effectively reverse this chemotherapy resistance [83]. Therefore, affecting the chemoresistance of BCSCs by regulating the occurrence of autophagy may be a novel option for breast cancer treatment.

## Summary

Autophagy exhibits distinct roles at different stages of breast cancer and other tumor development. The application of autophagy inhibitors or inducers during different stages of tumor development may be beneficial for both treatment and prevention. As the relationship between breast cancer and autophagy has been intensively studied, drugs related to autophagy for breast cancer treatment have shown clinical translational benefits. Autophagy inhibitors have emerged as novel anti-tumor agents, significantly expanding the spectrum of treatment options. The majority of data show that autophagy inhibitors are beneficial in the treatment of breast cancer, whether used in combination with cytotoxic chemotherapy, anti-estrogenic endocrine agents, or radiotherapy. For example, the regulatory mechanisms of autophagy in breast cancer are diverse, forming a complex regulatory network with interactions between different pathways and multiple influencing factors, which need to be further explored and recognized. The role of autophagy and its regulatory mechanisms can vary between different breast cancer subtypes and cancer cell differentiation states. Recognizing these differences is crucial for effectively applying these findings to both basic research and clinical translation. Therefore, revealing the role of autophagy in breast cancer and understanding its underlying mechanism will facilitate the transition from basic science to clinical applications. This knowledge serves as a basis for the combination of drug therapy for breast cancer patients in clinical settings, alleviating the burdens associated with traditional treatments.
